# Evaluation of Static Friction of Polycrystalline Ceramic Brackets after Conditioning with Different Powers of Er:YAG Laser

**DOI:** 10.1155/2015/749616

**Published:** 2015-09-27

**Authors:** Valiollah Arash, Saeed Javanmard, Zeinab Eftekhari, Manouchehr Rahmati-Kamel, Mohammad Bahadoram

**Affiliations:** ^1^Department of Orthodontics, Faculty of Dentistry, Babol University of Medical Sciences, Babol, Iran; ^2^Dental Materials Research Center, Babol University of Medical Sciences, Babol, Iran; ^3^Medical Student Research Committee & Social Determinant of Health Research Center, Ahvaz Jundishapur University of Medical Sciences, Ahvaz, Iran

## Abstract

This research aimed to reduce the friction between the wire and brackets by Er:YAG laser. To measure the friction between the wires and brackets in 0° and 10° of wire angulations, 40 polycrystalline ceramic brackets (Hubit, South Korea) were divided into 8 study groups and irradiated by 100, 200, and 300 mj/s of Er:YAG laser power. Two groups of brackets were not irradiated. The friction between the wires and brackets was measured with universal testing machine (SANTAM) with a segment of .019 × .025 SS wire pulled out of the slot of bracket. ANOVA and *t*-test were used for analyzing the results. To evaluate the effect of the laser on surface morphology of the bracket, SEM evaluations were carried out. The mean frictional resistances between the brackets and wires with 0° of angulation by increasing the laser power decreased compared with control group, but, in 10° of angulation, the friction increased regardless of the laser power and was comparable to the friction of nonirradiated brackets. Furthermore, with each laser power, frictional resistance of brackets in 10° of angulation was significantly higher than 0° of angulation. These results were explained by SEM images too.

## 1. Introduction

In the mid-1980s the first brackets made of monocrystalline ceramic (Sappire) and polycrystalline ceramic were marketed [1]. These brackets had many advantages compared with other esthetic appliances, including higher strength, more resistance to wear and deformation, more color stability, biocompatibility, and, above all, better appearance that is most demanded by patients during treatment nowadays. Ceramic brackets have some disadvantages, too, such as enamel wear, brittleness, difficulty debonding, and high coefficient of friction, which result in more resistance to sliding of wire in the slot [2].

It has been shown that, under all test circumstances, ceramic brackets have more frictional resistance compared with metal brackets because of their higher surface roughness. This concept is well ascertained by comparing these two brackets under a scanning electron microscope [3]. During orthodontic movement more over 60% of the force applied to teeth may disappear due to frictional resistance of ceramic brackets [4]. Higher frictional resistance increases the orthodontic force needed to move the teeth [5].

Reinforcing ceramic brackets with SS, improvement of manufacturing process of ceramic brackets, insertion of bumps on the floor of the slot of brackets, and so forth have been tried to reduce different problems of these brackets, including friction, strength, and force control [2, 4, 6–8]. However, investigations have shown inefficacy of some of these methods [4, 9].

Few researches have been carried out in orthodontics on the reduction of frictional resistance of ceramic brackets by laser. Abdallah et al. reported that glazing In-Ceram Alumina dental ceramics with high power settings of CO_2_ laser and high energy density of excimer (XeCl) laser can improve the surface hardness and smoothness of ceramic surfaces [10]. Kara et al. and Dilber et al. reported that application of Er:YAG and Nd:YAG lasers changed the surface roughness of ceramic blocks used in their study [11, 12]. Considering the results of these studies, this study aimed to reduce the friction between the wire and brackets by different powers of Er:YAG laser to improve surface characteristics of ceramic brackets.

## 2. Materials and Methods

**Figure 1:**
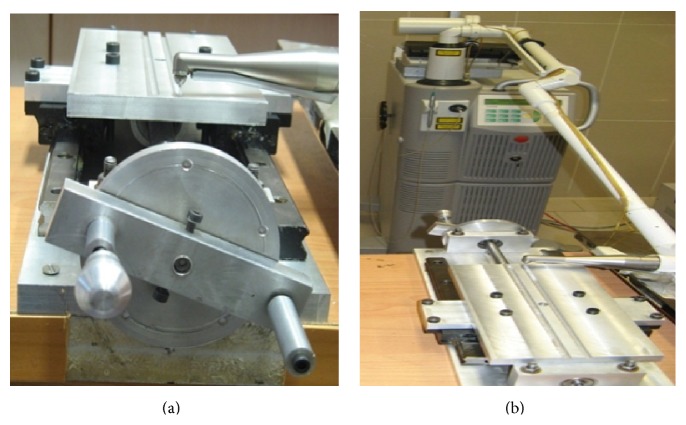
The table was designed to keep the laser sample distance, laser speed, and angle of laser ray constant. The brackets were placed on a slot propped on the table. The laser handpiece-bracket distance was 1 mm for all the samples.

In this experimental study 40 polycrystalline ceramic brackets (Hubit, South Korea) and 40 pieces of .019 × .025-inch stainless steel wire (Orthotechnology, USA) were used to measure the friction between the wires and brackets in 0° and 10° of wire-bracket angulations. The brackets were randomly divided into 8 study groups, each group including 5 brackets; 6 groups were irradiated with 100, 200, and 300 mj/s of Er:YAG laser with wavelength 2940 at room temperature. Therefore, for each power setting and in each wire-bracket angulation, 5 brackets were used. Two groups of 5 brackets in 0° and 10° served as controls for friction test and were not irradiated. The brackets were placed on a designed table under a fixed laser handpiece. The table was aimed at keeping the laser sample distance, laser speed, and angle of laser ray constant. The handpiece light fiber diameter was 0.9 mm, so the table was designed so that a screw could move the table 0.9 mm in each turn and move the bracket with it at the same distance (Figure 1). Bracket irradiation was carried out for 20 seconds on each 0.9 mm of bracket surface from a distance of 1 mm from the brackets to apply the maximum power of the laser.

### 2.1. Evaluation of Bracket Surface

The effect of the laser on the surface morphology of the brackets was evaluated under a scanning electron microscope (model KYKY-EM3200).

### 2.2. Friction Test

**Figure 2:**
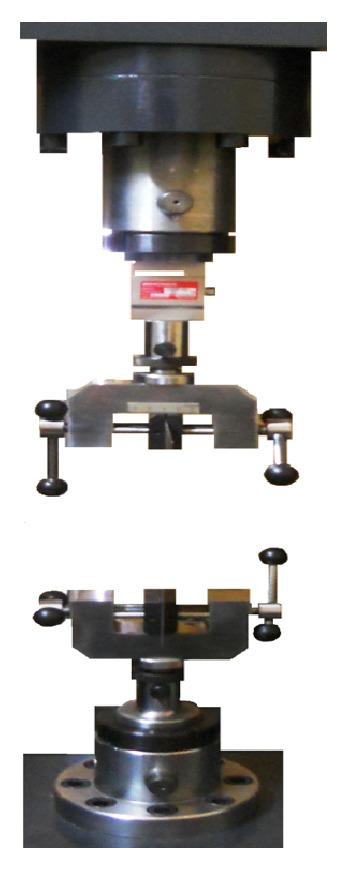
Schematic of universal testing machine. To measure the friction between the wire and bracket, a universal testing machine (SANTAM) (Sahand Company, Iran) was used. Straight line static traction test was carried out by this machine to simulate sliding of wire in the bracket. At one end of the machine there was an aluminum device that was attached to a 50 N load cell; this machine included two plates. The brackets were bonded to the lower stable plate. The wire was attached to the upper plate and then engaged in the bracket by elastomeric ligation; the wire was pulled out of the slot of the bracket with 50 N load cell at a rate 1 mm/min.

To measure the friction between the wire and bracket, a universal testing machine (SANTAM) (Sahand Company, Iran) was used. Straight line static traction test was carried out by this machine to simulate sliding of wire in the bracket. At one end of the machine there was an aluminum device that was attached to a 50 N load cell; this device included two plates. The brackets were bonded to the lower stable plate; then artificial saliva was sprayed on them. The wire was attached to the upper plate and then engaged in the bracket by elastomeric ligation. The upper end of the wire was attached to the upper plate; the wire was pulled out of the slot of the bracket with 50 N load cell at a rate 1 mm/min (Figure 2).

To place the brackets on the plate at 0° of angulation, the slots of the brackets were aligned precisely parallel to the edge of the plate by a conveyor (the edges of the plate were perpendicular to each other). To place the brackets on the plate at 10° of angulation, a line was drawn with 10° of angulation to the edge of the plate and the brackets were attached to the plate so that their slots were parallel to the line. The data were analyzed with ANOVA and *t*-test using SPSS 20.

## 3. Results

**Figure 3:**
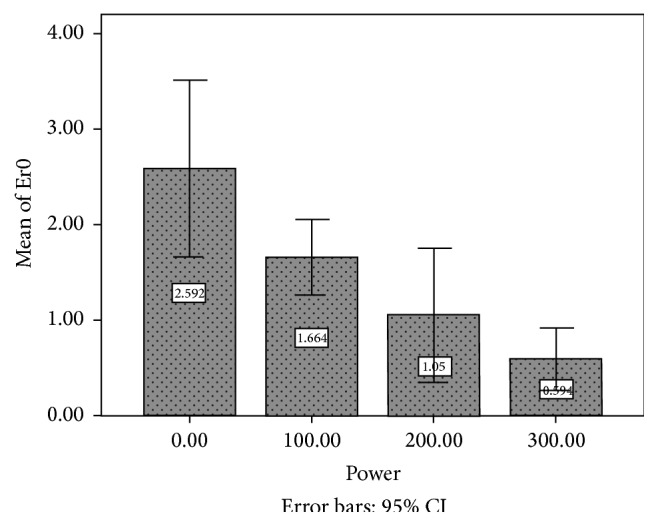
This diagram shows the changes in frictional force (N) between the wire and bracket at 0° of angulation with an increase in laser power (mj/s). Frictional resistance decreased with an increase in Er:YAG laser power (*P* = 0.000).

Table 1 and Figure 3 show the results of friction test between the wires placed at 0° of angulation and brackets irradiated with different laser powers.

As shown in Figure 3, irradiation of brackets with 100 mj/s laser power and an increase in the power to 300 mj/s resulted in a significant decrease in frictional resistance compared with the control group (*P* = 0.000).

The differences between the frictional resistance of samples irradiated with 100 mj/s and 200 mj/s were not significant (*P* = 0.264). In addition, there were no significant differences in the frictional resistance between samples irradiated with 200 mj/s and 300 mj/s (*P* = 0.508). However, the differences between the frictional resistance of the samples irradiated with 100 mj/s and 300 mj/s laser power were significant (*P* = 0.020).

**Figure 4:**
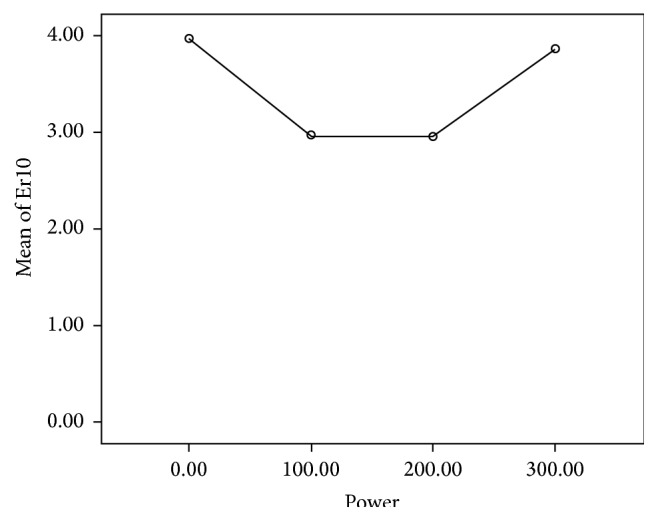
This diagram shows the changes in frictional forces (N) between the wire and bracket at 10° of angulation of wire with an increase in the laser power (mj/s).

Table 2 and Figure 4 show the results of friction test between the wires placed with 10° of angulation and brackets irradiated with different laser powers.

The changes in frictional resistance between the bracket and wire with an increase in the power of Er:YAG laser was not statistically significant (*P* = 0.397).

As shown in the diagram, irradiation of the brackets with 100 mj/s laser and an increase in the power to 300 mj/s did not result in statistically significant changes in frictional resistance compared with the control group.

The differences between the frictional resistance of the samples irradiated with 100 mj/s and 200 mj/s (*P* = 1.000); 200 mj/s and 300 mj/s (*P* = 0.642); 100 mj/s and 300 mj/s (*P* = 0.649) were not significant.

Table 3 shows the comparisons between the results of friction test between the wires placed at 10° of angulation and 0° of angulation and brackets irradiated with different laser powers. As shown in Table 3, with each laser power, the frictional resistance at 10° of angulation was statistically higher than frictional resistance at 0° of angulation with all laser powers.

## 4. Discussion

Friction control is a major challenge in orthodontic treatment since a part of the applied force is dissipated to overcome friction [13]. In this study we aimed to reduce the frictional force between ceramic brackets and wires by irradiation of Er:YAG laser beams on ceramic brackets. When we have adequate temperature and time for atomic displacement on the surface the sharp angles of grains tend to become rounding (atomic displacement is the process of movement of atoms from rough and bumpy regions to depressed regions) [14].

**Figure 5:**
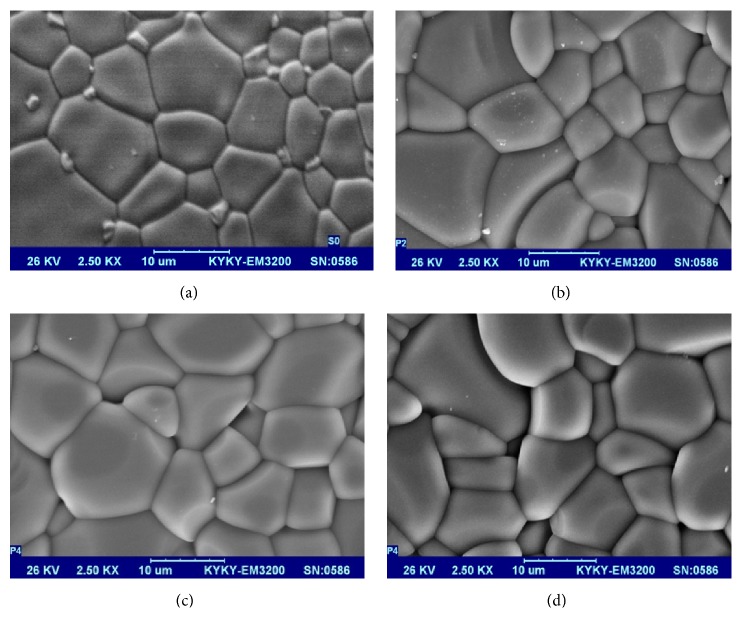
SEM images of brackets irradiated with 0, 100, 200, and 300 mj/s laser powers.

As seen in Figure 5, by increasing the laser intensity, the temperature increases and more intergranular melting and grain contraction happen; the result is rounded angles of grains. Of course for more accurate evaluation of surface morphology to determine the relation between friction and surface changes, atomic force microscope (AFM) imaging is needed.

On the other hand, there was no significant difference between frictional resistance of brackets irradiated with different laser powers and wires placed in 10° of angulation, which might be attributed to more impressive effect of binding (deflection of wire against the corners of bracket) compared with surface friction in producing frictional resistance when the wire is placed with an angle in the bracket. As the angle between the wires and brackets in all the samples of this group was the same regardless of the laser power and the angle between the wire and bracket determines the binding rate, the frictional resistances between these brackets were not significantly different from each other. Therefore when binding of wire against the corners of brackets occurs the surface friction between the wire and the floor of the slot does not represent frictional resistance [15]. This can also explain the higher frictional resistance of samples irradiated with each laser power with 10° angle of wire in the bracket compared with 0° angle.

Jones and Amoah compared the static frictional resistance of ceramic brackets with a conventional slot (Allure), a glazed slot (Mystique), and a metal slot insert (Clarity) in three different simulated binding angulations (0°, 5°, and 10°) for each type of bracket. They concluded that brackets with glazed slot demonstrated low frictional resistance at 0° of angulation (without binding) and their friction was comparable to a metal slot in a ceramic bracket; however, by increasing angulations from 5 to 10 degrees frictional resistance increased so that the bracket behaved more like a conventional polycrystalline ceramic bracket [16]. The results of this study, especially about the prominent effect of binding on frictional resistance, are consistent with our results.

Abdallah et al. reported that glazing In-Ceram Alumina dental ceramics with high power settings of CO_2_ laser and high energy density of excimer (XeCl) laser improved the surface hardness and smoothness of ceramic surfaces without affecting their internal structures [10]. Also Folwaczny et al. reported a significant reduction in roughness of dental ceramic surfaces under 308 nm excimer laser irradiation [17]. Zum Gahr et al. treated the surface of alumina with two procedures, followed by melting the surface with 200 W CO_2_ laser; friction coefficient of ceramic decreased as a result [18]. These results are also consistent with our results.

Since frictional force is caused by several factors, which are usually correlated and dependent, these factors can also influence and create undesirable behavior in the frictional force values. Moreover, it is difficult to compare studies because of different methodologies; these variables can be considered influencing factors in data registers. Intraoral variables such as saliva, plaque, acquired pellicle, corrosion, chewing, bone density, tooth number, anatomic configuration, root surface area, and occlusion were not evaluated in this study, but they can influence frictional force values. In vitro studies do not correspond to what really happens during dental movement, and, therefore, the reader must be careful when evaluating the results from this research. The friction magnitude recorded is substantially different from the applied forces in clinical orthodontic movement. The values recorded should be used to compare the effects of different factors rather than to quantify in vivo friction.

## 5. Conclusion

By increasing the power of laser beams applied to the brackets, the friction between the wire and bracket at 0° of angulation decreased; but, at 10° of angulation, the friction increased regardless of the laser power and was comparable to the friction of nonirradiated brackets.

## Figures and Tables

**Table 1:** 

Power of Er:YAG laser irradiated to samples (mj/s)	Average of frictional force between wire and bracket (N)
0	2.59 ± 0.74
100	1.66 ± 0.31
200	1.05 ± 0.56
300	0.59 ± 0.25

**Table 2:** 

Power of Er:YAG Laser irradiated to samples (mj/s)	Average of frictional force between the wire and bracket (N)
0	3.97 ± 0.75
100	2.97 ± 1.02
200	2.9 ± 1.66
300	3.8 ± 1.18

**Table 3:** 

Power of Er:YAG laser beams used (mj/s)	Means of frictional forces between the wire at 0° of angulation and the bracket (N)	Means of frictional forces between the wire at 10° of angulation and the bracket (N)	*P* value
0	2.59 ± 0.74	3.97 ± 0.75	0.020
100	1.66 ± 0.31	2.97 ± 1.02	0.026
200	1.05 ± 0.56	2.9 ± 1.66	0.041
300	0.59 ± 0.25	3.8 ± 1.18	0.000
